# Coupled dopamine and insulin signaling mediated transgenerational and multigenerational inheritance of adaptive traits in *Caenorhabditis elegans* upon parental training with *Salmonella enterica* Serovar Typhi

**DOI:** 10.1128/spectrum.02575-24

**Published:** 2025-05-22

**Authors:** Bynedi Seshadhri Chinna Mounish, Balasubramanian Chellammal Muthubharathi, Thirumugam Gowripriya, Koilmani Emmanuvel Rajan, Krishnaswamy Balamurugan

**Affiliations:** 1 Department of Biotechnology, Alagappa University 546926, Karaikudi, Tamil Nadu, India; 2 Behavioural Neuroscience Laboratory, Department of Animal Science, School of Life Sciences, Bharathidasan University 99006, Tiruchirappalli, Tamil Nadu, India; Institute of Microbiology, Chinese Academy of Sciences, Beijing, China

**Keywords:** Transgenerational inheritance, Multigenerational adaptations, Dopamine signaling, Neuronal plasticity

## Abstract

The nervous system’s ability to perceive and learn about the environment can help organisms evolve and acquire traits, potentially generating adaptive responses. However, its potential to produce heritable modulations is a scientific lacuna, which is under-explored. Here, with the help of *Caenorhabditis elegans*, which has a well-established neuronal networking, we found that on training the worms on a candidate pathogenic bacterium *Salmonella enterica* Serovar Typhi, the worms could exhibit a characteristic transgenerational pathogenic avoidance up to three subsequent generations to the otherwise attractive pathogen. Our further analyses suggested that dopamine signaling is essential for the learning and transmission of the learned traits across generations and that inhibiting or mutating the expression of DAT-1 involved in dopamine transportation eliminated the inheritance patterns. Also, the offspring generations showed enhanced survival resistance against *S*. Typhi, which was coupled with the higher levels of C-type lectins suggesting priming of the offspring’s immune system to generate resistance against *S*. Typhi upon re-exposure. Enhanced DAF-2/DAF-16-mediated insulin signaling pathway was observed, suggesting that the inherited immune response could be mediated through insulin/IGF-1 signaling (IIS). Furthermore, mutigenerational training on *S*. Typhi for three continuous generations induced preferential adaptation and better survivability toward *S*. Typhi. Taken together, the present study indicates that *S*. Typhi infection could generate transgenerational heritable dopaminergic modulations, which could possibly be the key signaling player in determining the decision-making ability of the host and also generate adaptive survival response, which could be mediated by the insulin-signaling pathway.

**IMPORTANCE:**

Adaptation is a phenomenon by which an organism learns and develops a mechanism to respond to dynamic and challenging conditions. It provides animals with an advantage to exhibit phenotypic as well as genotypic plasticity, enabling better survivability. The current study helps in understanding how animals respond to environmental stresses such as bacterial infections and the possible mechanism by which the information of the experience is being transmitted across future generations. Neuronal signaling promotes the brain’s ability to learn and generate memory, thereby reorganizing the response of the organism. The study also tries to understand how neuronal signaling could be essential for transmitting the information of parental experiences transgenerationally. Collectively, the study helps us understand the evolutionary adaptations exhibited across generations, which will also help us understand the long-term effects of pathogenesis.

## INTRODUCTION

Many organisms have developed defense strategies to adapt to the persistent threat of infections in a way that parental experiences can prime offspring immunity (1–3). Although August Weismann in the late 1800s proposed the “Weismann barrier” stating that the hereditary continuum is mediated only by germ cells and the soma, which is segregated during embryonic development, has no role in the induction of inheritance of traits, there is accumulating evidence to suggest that the Weismann barrier is not impermeable and that several somatic cell modifications can be inherited across generations (4, 5). The experiences, which induce heritable priming, could be several types ranging from food supply, heat stress, osmotic stress, bacterial infections, and mitochondrial stress (6–10). However, it is not still clearly known as to how the information is exactly passed from parent to offspring. Over the years, studies on inheritance mediated by chromatin alteration through epigenetic players have gained pace, owing to the use of model systems such as mice, *Drosophila melanogaster,* and *Caenorhabditis elegans* (11–13). Also, the ability of the nervous system to perceive information about the environment has been conserved across species. These mechanisms are involved in processing the external and internal physiological states to generate an adaptive response. However, whether the neuronal activity can be inherited is still a prime question waiting to be answered.

We use *C. elegans* as a model animal to study the bacterial pathogen-mediated modulations and the host’s response against the invading pathogen. During bacterial infection, *C. elegans* can generate a multifactorial immune response as a mechanism of resistance mediated by several conserved immune regulatory signaling pathways such as DAF-2/DAF-16 insulin signaling pathway, TGF-β signaling pathway, p38/PMK-1 MAP-Kinase signaling pathway, and Notch signaling pathway (14, 15). The insulin signaling pathway in *C. elegans* plays a very critical role in regulating longevity, metabolism, and stress responses including immunity. Mutations in *daf-2*, which encodes for Insulin/IGF-1 receptor, have been reported to enhance the resistance during *Pseudomonas aeruginosa* and *Staphylococcus aureus* infections. This is associated with the production of antimicrobial peptides and activation of downstream stress response pathways (16, 17). In addition, *C. elegans* has a well-structured neuronal network that allows it to sense danger in its vicinity and modulate its olfactory response to avoid the pathogen. This form of learning is mediated by biomolecules of the pathogen that help the worms to generate an adaptive mechanism, thereby escaping from the dangers of the pathogenic infection (18). Neurotransmitter signaling such as dopaminergic, serotonergic, and cholinergic plays a major role in the decision-making ability of *C. elegans* (19). Mutations in these regulating pathways have been reported to reduce the animal’s ability to learn and generate protective responses (20). It remains to be understood whether the neuronal regulation of pathogenic avoidance can be heritable in modulating the behavior of the offspring.

When a trait acquired upon environmental learning is transmitted to more than one generation, it is termed transgenerational inheritance. In *C. elegans,* transgenerational inheritance has been found to be regulated by epigenetic mechanisms such as RNAi pathways, histone modifications, and chromatin modifiers (18, 21, 22). Exposure to *P. aeruginosa* has been reported to induce pathogenic avoidance across multiple generations through Piwi/PRG-1-mediated epigenetic modulations (23). In another study, starvation of *C. elegans* induced the generation of small RNAs across multiple generations which were found to be involved in conferring enhanced nutritional levels in the offspring (24). The effect of transgenerational inheritance might not be uniform across infections and might vary, depending on the condition. The heritable effects might be dynamic in response, depending on the type of stress. Flock House viral infection induced the production of viral small RNA-based anti-viral response to silence the viral genome, which was transmitted across generations to generate an adaptive response in offspring (25). Concurrently, another research group showed that the RNAi machinery involved in rendering immunity against Orsay viral infection was not observed to be transgenerational and was limited to the infected generation alone (26). These studies suggest that the heritable effects might be dynamic in response, depending on the type of stress. Hence, there is a need to understand the diverse mechanisms of transgenerational effects across different forms of stress. Despite all the advances made in the field, there is still a larger scope left to study the role of sensory players such as neurotransmitter-mediated signaling across multiple generations during different conditions.

In the present study, by using *S*. Typhi as a candidate pathogen to train *C. elegans* for a limited duration, we have studied the long-term transgenerational effects of their offspring. Although earlier studies have highlighted the effects of *S*. Typhi infection and the induction of host immune response as a combating strategy, the long-term transgenerational effects of the parental infection would help us understand how organisms evolve to overcome a stressful condition during subsequent generations (15, 27, 28). The present study focuses on answering the following questions: (i) How does parental exposure to pathogenic bacterium modulate the response of the subsequent offspring/generations? (ii) If there was a modulated response generated, how long that response can persist and which molecular players are responsible for it? In addition, we also wanted to find out what would be the response when the *C. elegans* were exposed to the same pathogen for multiple continuous generations.

## MATERIALS AND METHODS

### *C. elegans* and bacterial strains maintenance

The wild-type N2 Bristol strain *C. elegans* along with the transgenic strains BZ555; egIs1 [p*dat-1*::GFP] and the mutant strain RM2702; *dat-1*(ok157) III were obtained from Caenorhabditis Genetics Center (CGC), University of Minnesota, USA. The nematodes were maintained under standard conditions on Nematode Growth Media (NGM) at 20°C and fed with *Escherichia coli* OP50. For training, plates with the respective bacterial culture were spread over the NGM plate as a full-lawn. The plates were incubated at room temperature overnight to attain a uniform growth across the medium. Furthermore, the plates were stored at 4°C until required. For all experiments, synchronized L4 stage worms were obtained by lysing the gravid adult worms with an alkaline sodium hypochlorite solution containing 5 M potassium hydroxide (KOH) in a ratio of 1:1 (27). The bacterial strain *S*. Typhi (MTCC 733) (ST) was obtained from MTCC (Microbial Type Culture Collection, IMTECH, Chandigarh, India). The strains *Vibrio alginolyticus* (ATCC 17749) and *Lactobacillus plantarum* (ATCC 14917) were obtained from (American Type Culture Collection, USA). *E. coli* OP50, *S*. Typhi, and *Lactobacillus plantarum* were maintained in an LB medium at 37°C. *Vibrio alginolyticus* was maintained in Zobelle Marine medium at 30°C. For all the experiments, ∼0.2 OD at 600 nm of bacterial cultures was used. All the assays were performed in biological triplicate.

### Transgenerational and multigenerational bacterial training

**Fig 1:**
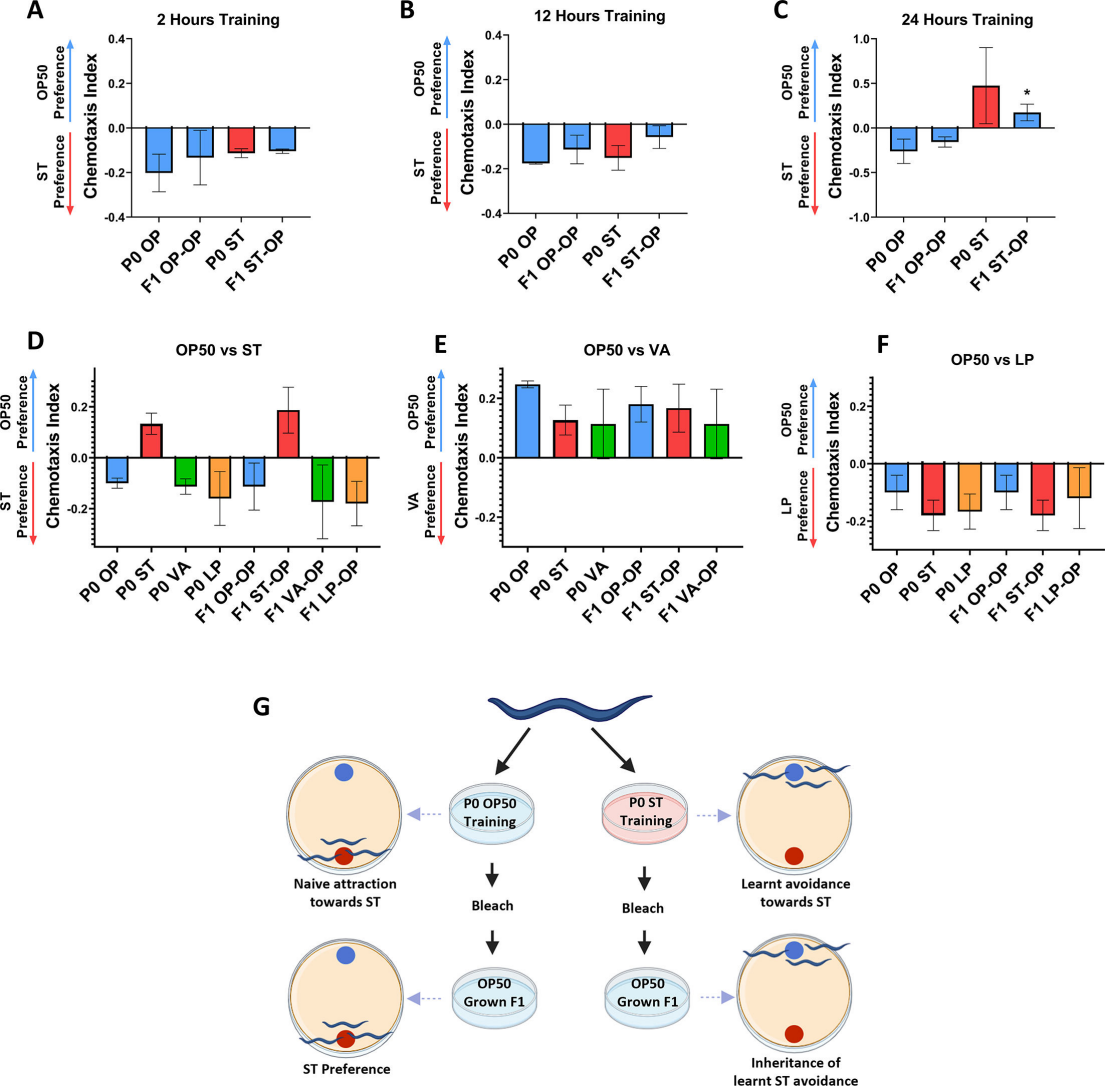
Heritable modulations of *S*. Typhi training of *C. elegans* in P0 parental generation. Chemotaxis assay analysis of *C. elegans* trained with *S*. Typhi and their F1 offspring for 2 h (**A**), 12 h (**B**), and 24 h (**C**), *n*= ~450 worms, (Blue color corresponds to *C. elegans* grown on *E. coli* OP50 before experimentation, and red color corresponds to *C. elegans* grown on *S*. Typhi before experimentation). Specificity of learned pathogenic avoidance of *S*. Typhi, *V. alginolyticus,* and *L. plantarum*-trained *C. elegans* parent worms and their F1 offspring against *S*. Typhi (**D**), *V. alginolyticus* (**E**), and *L. plantarum* (**F**)*, n* = ~450 worms. (**G**) Schematic representation of heritable learned pathogenic avoidance behavior upon *S*. Typhi training of *C. elegans*.

P0 parent L4 stage worms (approximately 300 worms on a 3 cm bacterial lawn) were washed thoroughly with M9 buffer and placed on the *S*. Typhi training plates. These worms were termed P0 OP (*E. coli* OP50-trained parents) and P0 ST (*S*. Typhi-trained parents). The plates were incubated at 20°C for 24 h. After training, the worms were washed thrice with M9 buffer to remove the residual bacteria. Approximately half the numbers of the worms were used for experiments, whereas the remaining worms were used to obtain the F1 generation by bleaching the P0 parent worms as shown in Fig. 1G. For transgenerational experiments, only the P0 parent worms were trained on the pathogen, and the subsequent generations were grown on *E. coli* OP50. The offspring obtained from P0 ST-trained parent worms were grown in *E. coli* OP50. These worms were labeled as F1 ST-OP. The F1 ST-OP worms were used for experiments on day 1 adult stage. The remaining F1 ST-OP worms were bleached to obtain F2 generation, also grown on *E. coli* OP50 termed as F2 ST-OP. For multigenerational experiments, the F1 offspring worms of P0 ST parents were grown on *E. coli* OP50 until the L4 stage, and then, they were washed and exposed to *S*. Typhi for 24 h. These worms were termed F1 ST-ST. After training, the worms were used for experiments, and a part of the population was bleached to obtain F2 progeny, which were again re-exposed to the pathogen at L4 stage (F2 ST-ST). *E. coli* OP50-trained *C. elegans* were used as control for the experiments.

### Chemotaxis assay

The olfactory modulations of day 1 adult *C. elegans* post-infection toward the bacteria were analyzed by assessing its chemotactic preference and avoidance. Bacterial cultures (10 µL) at ∼0.2 OD (*E. coli* OP50 and *S*. Typhi) were spotted on either side as zone A and zone B in a 6 cm NGM plate, which wasin 20°C, and the number incubated at room temperature for 12 h prior to experimentation (29). The trained day 1 adult P0 parent/progeny worms were washed thrice with M9 buffer, and ∼50 worms were placed at the center of the plate, equidistant from the bacterial spots. The plates were kept at 20°C, and the number of worms at each spot was noted until 6 h. The preference or avoidance was calculated using a chemotaxis index toward *E. coli* OP50 (30).


$$\begin{array}{r} Chemotaxis\;Index=\frac{No.\;of\;worms\;at\;E.coli\;OP50\;spot-No.of\;worms\;at\;S.Typhi\;spot}{Total\;No.of\;worms\;assayed} \end{array}$$

The experiment was conducted in biological triplicates, and the average of the findings was used for plotting the graph, n = ~450 worms.

### Neuronal modulation

To understand the importance of neuronal signaling in *C. elegans* olfactory modulations, we used chemical interventions prior to bacterial exposure. Nisoxetine at 20 µM, scopolamine at 500 µM, and aldicarb at 1 mM concentrations were used to modulate dopamine signaling, serotonin signaling, and acetylcholine signaling, respectively (31–34). Briefly, synchronized L4 *C. elegans* were washed with M9 buffer and were exposed to the chemicals at the mentioned concentrations along with 20% bacterial inoculum, which was *E. coli* OP50, and incubated at 20°C for 3 h in a liquid medium (M9 buffer). After incubation, the worms were washed thrice with M9 buffer and subjected to bacterial training (*E. coli* OP50 and *S.* Typhi) and used for chemotaxis experiments.

### Bacterial colonization assay

To check the bacterial load inside the body of the worm, a colonization assay was performed. Briefly, the worms were washed thrice with M9 buffer after bacterial training and then treated with 100 µg/mL tetracycline to remove the bacteria adhered to its surface and in the buffer (35). Post-treatment, tetracycline was removed, and the worms were washed twice with M9 buffer to remove the tetracycline to avoid its interference with the internal bacteria inside the body upon ingestion. The worms (10 Nos.) were added to 100 µL of 1× PBS and were lysed for 30 s (10–10−10 s) with a mini-handheld homogenizer (Moxcare MT-13K, Haryana, India). The homogenate was diluted and spread across the respective medium. The selective media Salmonella Shigella agar was used for plating the worm homogenates. The number of colonies was counted manually after 16 h of incubation at their corresponding growth temperatures and colony-forming unit (CFU) was calculated.

### Survival assay

To assess the life span of *C. elegans,* a liquid medium-based survival assay was performed. The L4-staged worms were washed with M9 buffer, and ~10 worms were added per well to each group in 24-well plate consisting of 20% of bacterial culture and M9 buffer (36). Gametogenesis was inhibited by adding 50 µM 5-Fluoro-2-deoxyuridine (FuDR) to the wells. The number of worms surviving was recorded at regular intervals. The experiments were conducted in biological triplicates (n = ~90 worms for the assay), and the average was taken to plot the graph.

### Brood size assay

To assess the reproductive fitness of *C. elegans*, the number of progenies generated was counted. The L4 stage worms were washed, and 10 worms were added per plate for a group in triplicates on bacterial lawn NGM plates. The worms were transferred every 24 h to a new lawn plate of the same bacterium. The number of hatched worms was counted on the next day (37). The experiments were done in biological triplicates with a total of ~45 worms per condition. The progenies were counted across the lifespan, and the mean value was considered for plotting the graph.

### Brightfield microscopy analysis

To observe the morphological changes in the worm upon test bacterial training, brightfield microscopy analyses were done. Briefly, the worms were washed with M9 buffer and paralyzed using 1:100 ratio 100 mM sodium azide. Then, they were observed under a microscope with respective control groups with 10× objective magnification (38).

### Smurf assay

The intestinal barrier integrity was assessed using the Smurf assay as mentioned by (39). Briefly, after incubation on the respective bacterium, the worms were washed thrice with M9 buffer and suspended in a liquid medium (1:1) of M9 buffer mixed with blue food dye (5% Erioglaucine disodium salt in water) and incubated at RT for 3 h under in a shaker. Post-incubation, the worms were washed and paralyzed using 1:100 ratio 100 mM sodium azide. The worms were visualized for the presence or absence of leaked blue color from the intestinal lumen under a brightfield microscope with 10× objective magnification. Under healthy conditions (OP50 fed), the dye remains confined within the intestinal lumen, whereas upon pathogen colonization during infection, due to damage in the intestinal barrier, the dye leaks to the surrounding tissue turning the body of the worm blue.

### Fluorescent microscopy analysis

The neuronal expression of p*dat-1*::GFP was documented by using transgenic strains of *C. elegans* (BZ555). The worms were washed thrice with M9 buffer and were paralyzed. Then, they were fixed on a clean glass slide with the help of a coverslip and observed under a fluorescence microscope (Nikon Eclipse TS2R-C-AL, Tokyo, Japan). The gain was set at 200 ms under a 20× objective magnification for all the samples.

### RNA fluorescence *in situ* hybridization (FISH)

RNA fluorescence *in situ* hybridization was performed for visualization of the bacterial colonization inside the body of *C. elegans* by following the protocol mentioned in (40) with minor modifications. Briefly, the worms were collected in a sterile tube and washed thrice with 1× PBS + 0.1% Tween 20 (PBS-T) buffer by spinning the worm at 500 × *g* for 1 min. Then, the worms were fixed using 4% Paraformaldehyde (PFA) by adding 33 µL of already prepared 16% PFA to 100 µL of PBS-T buffer containing worms and incubated for 45 min at room temperature. After incubation, PFA was aspirated, and the worms were incubated overnight at 4°C in 70% ethanol. Then, ethanol was removed, and the fixed worms were gently washed to avoid breakage using PBS-T thrice by centrifugation. The worms were then washed once with the freshly prepared hybridization buffer (HB) (900 mM NaCl, 20 mM Tris pH 7.5, 0.01% SDS). After centrifugation, the buffer was removed leaving 100 µL of HB with worms to which the fluorophore-labeled FISH probes were added to attain a final concentration of 10 ng/µL. The samples were incubated in the dark for 16 h at 48°C in a water bath by gently mixing by tapping every 1 h. After incubation, the HB-containing FISH probes were removed, and the worms were washed with freshly prepared wash buffer (WB) (900 mM NaCl, 20 mM Tris pH 7.5, 5 mM EDTA, 0.01% SDS) and incubated at 51°C for 1 h by gently inverting the tube every 15–20 min. Then, the WB was aspirated, and the worms were stored PBS-T and then subjected to imaging using Confocal Laser Scanning Microscope (LSM 710, Carl Zeiss, Germany). The FISH probe used in the study is Sal3 (5′-AATCACTTCACCTACGTG-3′), labeled with fluorophore Cyanine 3 (Cy3) at its 5’ end, which binds specifically to 23S rRNA of *Salmonella* species (41). The gain of 720 ms and digital gain of 5.6 was set for all fluorescent images.

### Gene expression studies using transcriptomic analysis

The sample groups were washed with M9 buffer, followed by a final DEPC wash individually; 1 mL of TRIzol reagent (RNA X Press reagent, Himedia) was added to the worms, and the samples were frozen in −80°C for 24 h. The samples were then thawed and vortexed well prior to RNA extraction. The samples were centrifuged at 12,000 rpm for 15 min at 4°C where the obtained supernatant was collected and transferred to a fresh RNase-free tube. To the supernatant, 500 µL of ice-cold chloroform was added to induce phase separation. The samples were vortexed briefly and incubated in ice for 5 min, followed by centrifugation at 12,000 rpm for 15 min at 4°C. The aqueous phase was collected into a fresh tube, and 500 µL of ice-cold isopropanol was added and mixed gently. The samples were incubated at RT for 15 min, followed by centrifugation at 12,000 rpm for 10 min at 4°C. The obtained pellet was washed using 100 µL of 75% ethanol and centrifuged at 7,500 rpm for 5 min at 4°C. The RNA pellet was air-dried and resuspended with DEPC water. The isolated RNA was then reverse-transcribed using oligo dT primer and MultiScribe Reverse Transcriptase (Applied Biosystems) enzyme following the guidelines provided by the manufacturer. For assessing the expression of bacterial genes, Random Hexamer primer was used for cDNA conversion. For quantitative PCR studies, Bio-rad iTaq Universal SYBR Green Supermix was used to assess the expression of candidate genes as mentioned in Table 1. *act-2* was used as the housekeeping gene. The primers were designed using Prime3Plus online tool, and the experiments were carried out in triplicates. Polymerization and quantification were performed using real-time PCR instrument (Bio-rad CFX96 Touch Real-Time PCR Detection System). To quantify the fold change, the double delta Ct method was used (42).

**TABLE 1:** List of primers used in the study

Name of the gene	Forward primer	Reverse primer
*act-2*	5′-ACGCCAACACTGTTCTTTCC-3′	5′-TTCATGGTTGATGGGGCAAG-3′
*dat-1*	5′-TGGGCATCCTGTAACAACAGT-3′	5′-CAGCTGAGACAGATTGATTTGC-3′
*cat-2*	5′-TGAACGACGAAGGAATCGAA-3′	5′-GTCGTGTGAAGCCTCATTGC-3′
*daf-2*	5′-TCGAGCTCTTCCTACGGTGT-3′	5′-CATCTTGTCCACCACGTGTC-3′
*daf-16*	5′-ACCGTTCAAGCTGCTGC-3′	5′-AGGACGGCGGTTGTTGT-3′
*akt-1*	5′-ATGCGGAGTCGGCAGAA-3′	5′-TCACCGTGTCCCGAAGA-3′
*sgk-1*	5′-CGTTTTTACGCGGCCGA-3′	5′-TCCGGCGTCCCACAAAA-3′
*clec-60*	5′-ACAACAAAGCTGCGGCG-3′	5′-ACCCTTGTTTGCCGGCT-3′
*clec-67*	5′-TCTCCAATGTCCGTGCCA-3′	5′-AGCCCGCTGGTTGCATT-3′
*clec-87*	5′-AACGACGAACAGCCGCA-3′	5′-AGCACGAATCGGCGAAC-3′

### Statistical analyses

All the experiments were performed in independent triplicates. The experimental mean value was taken, the standard deviation was calculated, and the graph was plotted using GraphPad Prism 8.0. The significance was calculated using a one-sample *t*-test or two-way ANOVA (GraphPad Prism 8.0). The significance was represented as *P < 0.05 and **P < 0.01, ***P < 0.001 and ****P < 0.0001 in this study.

## RESULTS

### Parental training on *S.* Typhi can induce heritable learned pathogenic avoidance behavior

**Fig 2:**
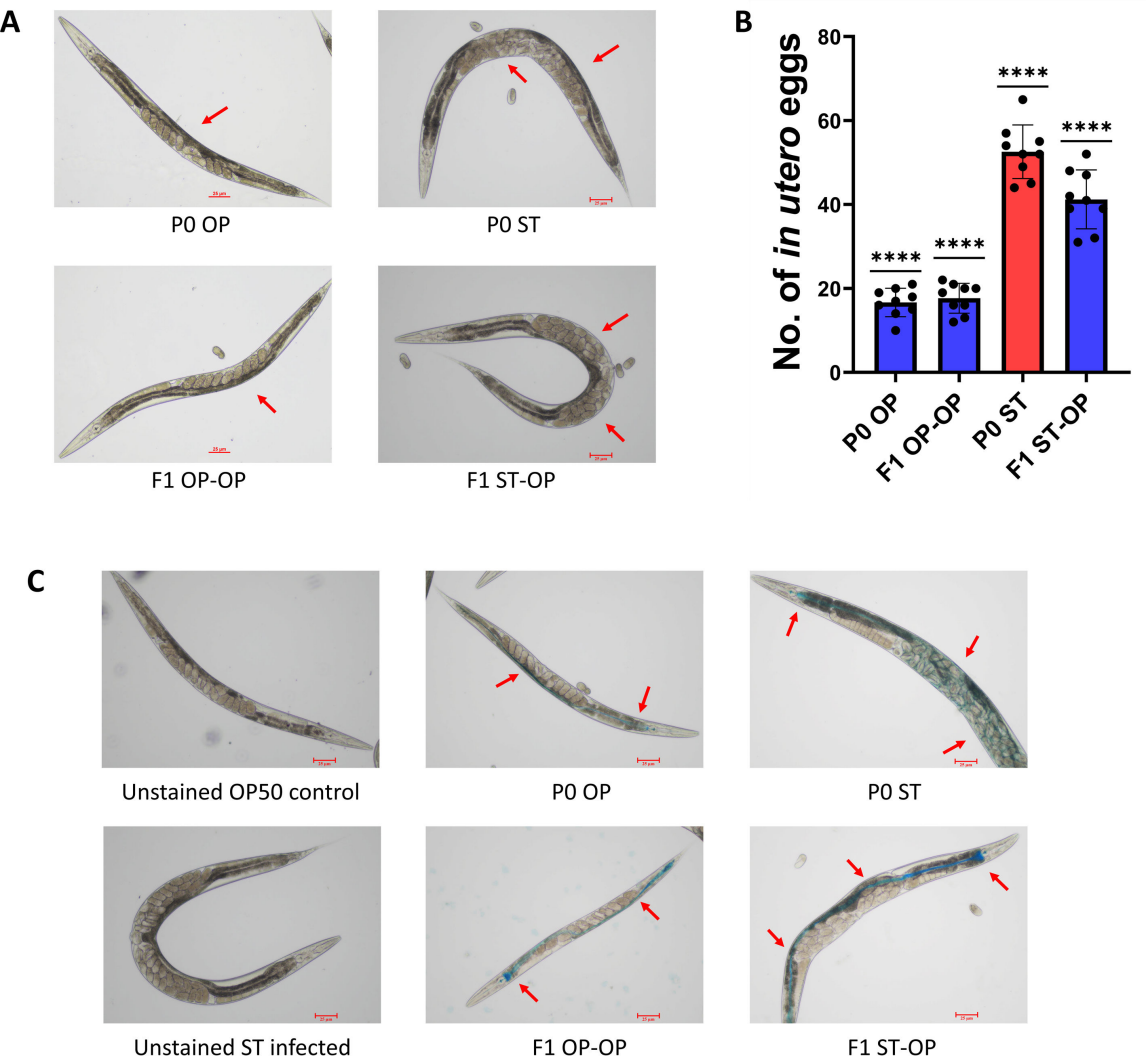
Morphological alterations in *C. elegans* trained with *S*. Typhi and their F1 offspring. (**A**) Brightfield microscopic analysis to assess the accumulation of eggs inside the uterus after 24 h of bacterial feeding (Scale bar: 25 µm). The red colored arrow marks represent the eggs in the uterus where in P0 ST and F1ST-OP, higher accumulation was observed. (**B**) Quantitative analysis of egg count inside the uterus, *n* = 9. The significance was calculated using one sample t test (*P* < 0.0001) and corresponds to the variation in the replicates within the group. (**C**) Assessment of intestinal lumen damage using a Smurf assay, where blue color corresponds to the absorption of dye in the intestine (Scale bar: 25 µm). The red-colored arrow marks correspond to the accumulation of the Erioglaucine dye in the body of *C. elegans* upon bacterial feeding. The presence of the dye beyond the intestine in P0 ST suggests a damaged intestinal barrier causing leakage in the lumen.

Previous reports from our lab reported that exposing *C. elegans* to the bacterium in the form of conditioning or training can alter its olfactory behavior (43). Also, *C. elegans* are found to exhibit learned avoidance behavior toward the *Pseudomonas aeruginosa* PA14 after a brief exposure to the pathogen (44). Therefore, to determine whether the nematodes exhibit learned avoidance toward the pathogenic bacterium *Salmonella enterica* Serovar Typhi, their olfactory preference for the bacteria was analyzed after being trained on pathogenic lawn plates for various durations (2, 12, and 24 hours). It was observed that the naive worms which were trained in *E. coli* OP50 preferred the pathogen *S.* Typhi but when the *C. elegans* were trained *S.* Typhi lawn for 24 hours, they learnt to avoid the pathogen during its exposure. Interestingly, it is also noted the avoidance exhibited by the P0 parent *C. elegans* was also displayed by its F1 offspring generation even when they were grown on *E. coli* OP50 (F1 ST-OP). This heritable learnt avoidance toward the pathogen was not observed when the *C. elegans* were trained for 2 and 12 h, suggesting that a continuous exposure of 24 h in the pathogen *S.* Typhi was required to generate a modulation in the olfactory behavior (Fig. 1A through C). Furthermore, to understand if this heritable avoidance behavior is specific toward *S.* Typhi, we checked the chemotactic preference of the worms against a gram-negative pathogen *Vibrio alginolyticus* and a gram-positive non-pathogenic bacterium *Lactobacillus plantarum.* Neither the P0 *S.* Typhi-trained *C. elegans* nor its F1 ST-OP offspring showed any modulatory olfactory behavior toward *V. alginolyticus* and *L. plantarum* and exhibited similar behavior as that of the *E. coli* OP50-trained parent and offspring (Fig. 1D through F). For comparison, the experiment was also carried out by training the worms on *V. alginolyticus* and *L. plantarum. C. elegans* has a natural avoidance toward *V. alginolyticus* and attraction toward *L. plantarum.* Hence, the parental worms trained on either of the bacterium (P0 OP, P0 ST, P0 VA, and P0 LP) along with their offspring (F1 OP-OP, F1 ST-OP, F1 VA-OP, and F1 LP-OP) elicited avoidance behavior when the olfactory modulation was checked against *E. coli* OP50 and *V. alginolyticus*, and attractive behavior when checked against *E. coli* OP50 and *L. plantarum* (Fig. 1E and F). These results suggest that training of *C. elegans* for 24 h on *S.* Typhi could induce certain heritable physiological and behavioral alterations in the form of learnt pathogenic avoidance, which is specific toward a pathogen on which the worms were trained (Fig. 1G). Also, the physiological modulation in the form of a higher accumulation of eggs in the germline was observed and quantified where 52 ± 6 eggs were observed in *S.* Typhi-trained P0 parent and 41 ± 7 were observed in their F1 offspring, which was higher than the *E. coli* OP50 fed parent and their offspring (16 ± 3 and 17 ± 3, respectively) (Fig. 2A and B). Furthermore, since pathogenic bacteria predominantly colonize the intestine, the integrity of the lumen was assessed using the Smurf assay. Under P0 OP conditions, the dye was observed within the intestine, but in P0 ST, the leakage in the dye was noted in the body due to the intestinal damage, which might be the reason for the transmission of the pathogen from P0 parent to F1 offspring (Fig. 2C).

### Bacterial transmission might not be the only factor influencing the transgenerational inheritance of learnt pathogenic avoidance

**Fig 3:**
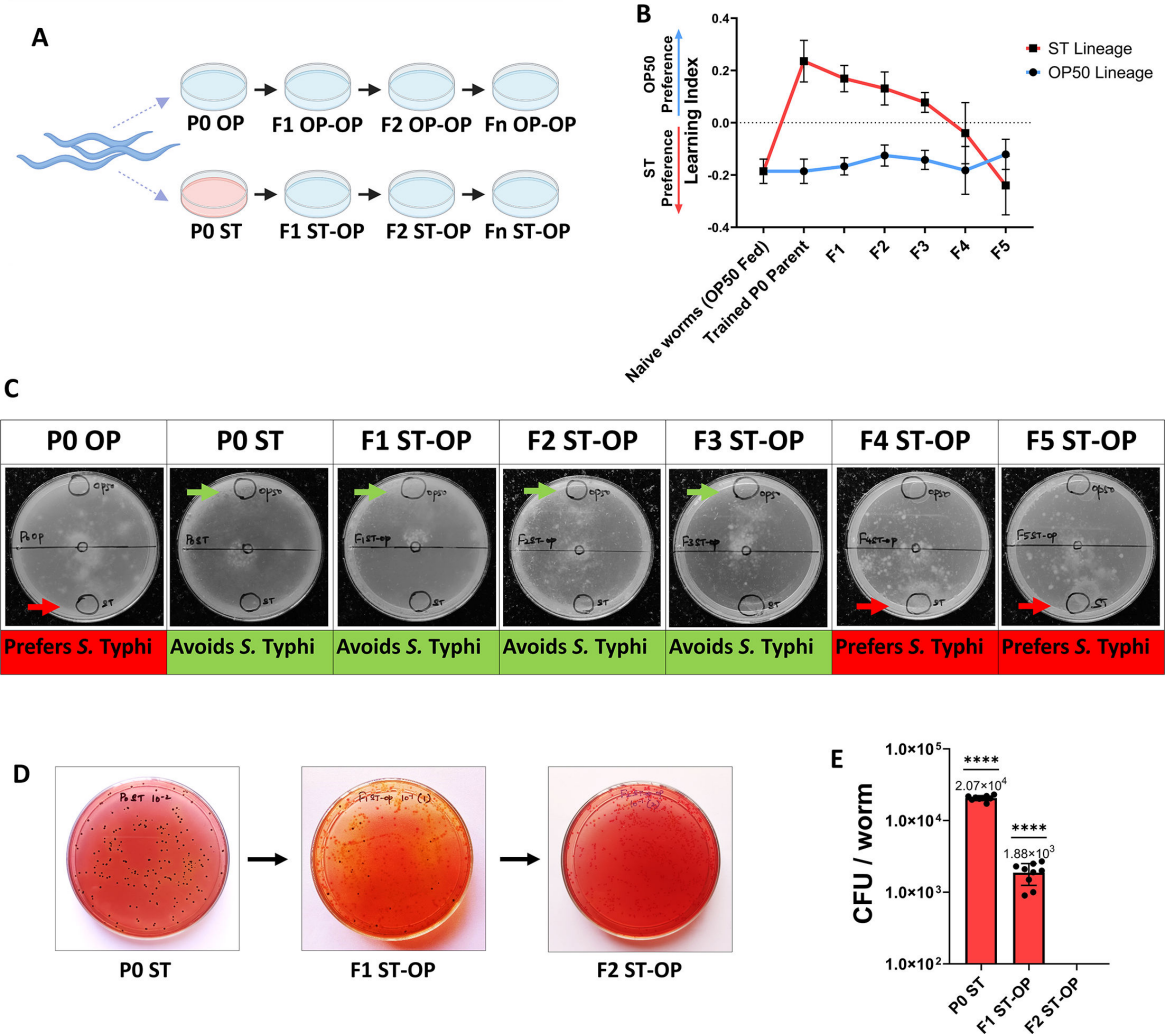
Transgenerational effects of *S.* Typhi training of P0 parental generation *C. elegans*. (**A**) Schematic representation of transgenerational experimental setup where the P0 parental generation is trained in *S.* Typhi and their subsequent generations are grown on laboratory food source *E. coli* OP50 (Blue color plate denotes *C. elegans* grown on *E. coli* OP50 and red color denotes *C. elegans* infected with *S.* Typhi in the P0 generation). (**B**) Transgenerational olfactory preference analysis of P0 *S.* Typhi-trained *C. elegans* and their subsequent generations (F1–F5), *n*= ~450 worms (Blue line corresponds to the lineage of *E. coli* OP50 fed P0 parent worms and red color corresponds to the lineage of *S.* Typhi-trained P0 parent worms). (**C**) Images of transgenerational olfactory preference analysis by chemotaxis analysis. The arrow marks correspond to the preferred bacteria in the condition. (**D**) Images of colonization assay of *S.* Typhi-trained P0 parent generation and the subsequent F1 and F2 generations, *n* = 10 worms, and dilution of 10^−2^ was used for P0 *S.* Typhi-trained worms and dilution of 10^−1^ was used for the offspring generations, and plates were prepared using *Salmonella Shigella* (SS) Agar. (**E**) Logarithm representation of colony-forming units calculated per worm.

**Fig 4:**
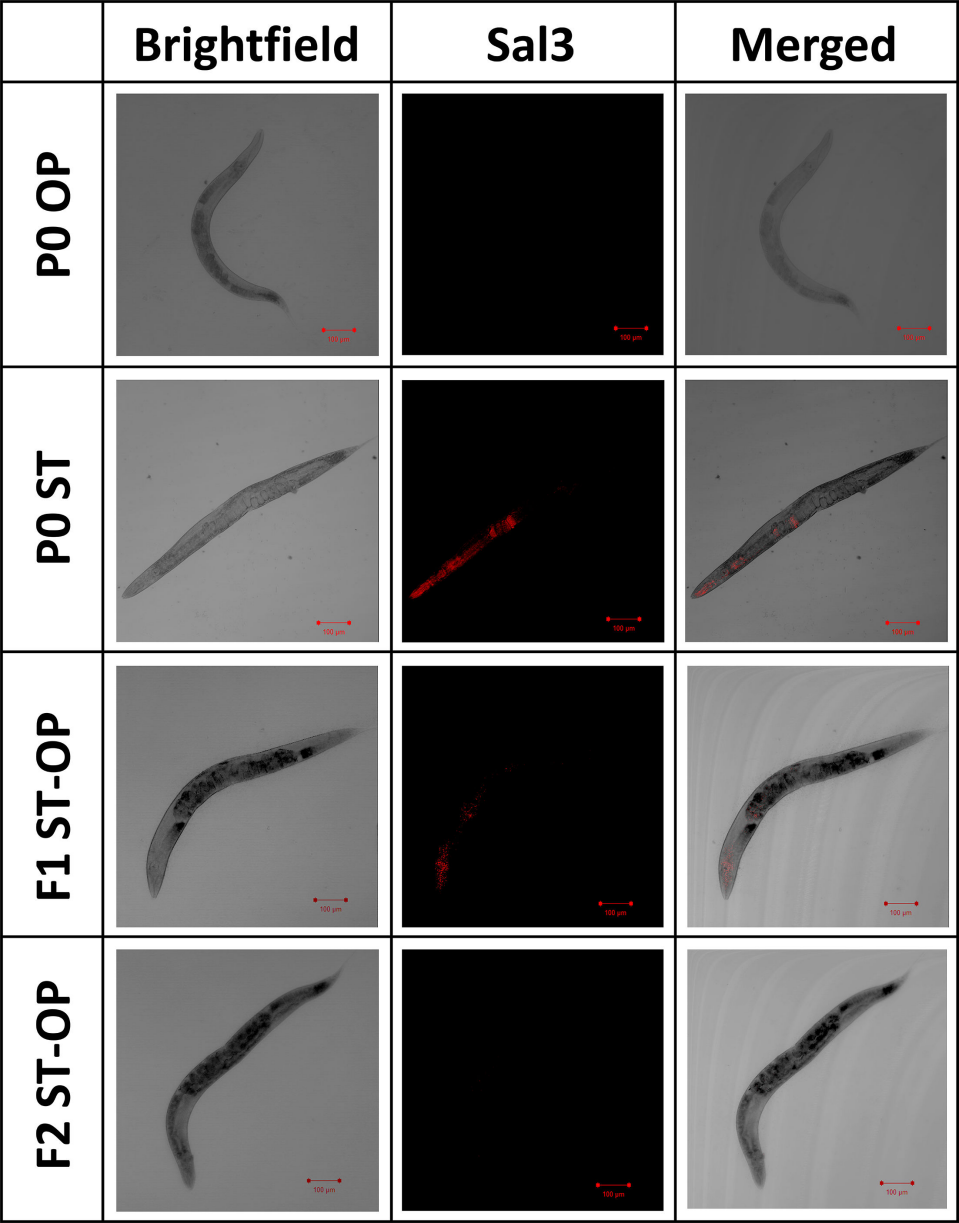
RNA fluorescence *in situ* hybridization (FISH) to visualize bacterial colonization inside the *C. elegans*. (Top to Bottom) Bacterial colonization was visualized transgenerationally upon *E. coli* OP50 fed control and *S.* Typhi-trained parent *C. elegans* and their subsequent offspring generations. (Left to Right). The images were captured in Brightfield mode, hybridization using Cy3-labeled Sal3 probe (*Salmonella* specific 23S rRNA probe; Red-colored fluorescence), and the merged images, (Scale bar: 100 µm).

To understand the number of generations the avoidance behavior persists upon P0 training on *S.* Typhi, the olfactory preference of the offspring was further analyzed transgenerationally from F1 to F5 generations (Fig. 3A). It was observed that the learnt avoidance toward *S.* Typhi was exhibited by the three consecutive offspring generations (F1 ST-OP, F2 ST-OP, and F3 ST-OP) of the P0 *S.* Typhi-trained *C. elegans.* The F4 ST-OP and F5 ST-OP generation *C. elegans* started to prefer the pathogen, suggesting that the effect is transient and fades off after F3 generation (Fig. 3B and C). As the vertical transmission of bacteria from one generation to another was reported from previous work in our lab (45), we checked if the observed transgenerational learnt pathogenic avoidance behavior was due to the presence of bacteria that colonized the worm by using bacterial colonization assay; ~10 adult *C. elegans* from each generation including the P0 *S.* Typhi-trained and their consecutive offspring generations on *E. coli* OP50 were examined for the presence of the *S*. Typhi accumulation in the body. The bacterial colonization of *S*. Typhi in the worms is 2.07*10^4^ CFU/worm in *S.* Typhi-trained P0 generation and 1.88*10^3^ CFU/worm *S.* Typhi cells in the F1 ST-OP generation, which was obtained by bleaching the P0 parent antibiotic treatment to remove bacterial cells adhered to the body. Interestingly, we did not observe the presence of *S*. Typhi colonies from F2 ST-OP generation (Fig. 3D and E). Since the F1 and F2 were grown in *E. coli* OP50, which was devoid of *S.* Typhi, there were a large number of *E. coli* OP50 colonies that appeared as pink colonies in SS agar media. Furthermore, to confirm the transmission of the pathogen from the parent to the offspring, colonization assay was also performed under other parameters as bleaching without antibiotic treatment, allowing the P0 parent worms to naturally lay eggs after antibiotic treatment and without antibiotic treatment where the result showed 2.12*10^3^ CFU/worm, 2.69*10^3^ CFU/worm, and 3.02*10^3^ CFU/worm *S.* Typhi cells in the F1 offspring, respectively. Also, to check if vertical transmission is a common phenomenon observed across bacterial species, we checked the colonization of P0 and F1 upon training with *V. alginolyticus* and *L. plantarum*. Our results showed that the transmission of the bacterium from parent to offspring in *C. elegans* is observed when the parents were trained in *V. alginolyticus* and *L. plantarum also,* suggesting that it is a common phenomenon. Also, to visualize the bacterial colonization and transmission within *C. elegans*, RNA fluorescence *in situ* hybridization was carried out by hybridizing the worms with a fluorescent tag-labeled Sal3 probe specific to 23S rRNA of *S.* Typhi. The images in Fig. 4 show *S.* Typhi accumulation near the pharynx and the intestinal of the P0 parent worms trained on *S.* Typhi. In the F1 ST-OP worms, reduced accumulation of *S.* Typhi was observed, similar to the results of the colonization assay. Thus, *S.* Typhi colonization was only inherited for one generation (i.e., F1). These results suggest that olfactory response was generated even in F1–F3 generations, which were devoid of pathogenic colonization from vertical transmission. Therefore, upon the training of *C. elegans* in *S.* Typhi, pathogenic colonization might generate a memory of the exposure, which could be transmitted and would be required for the modulation of the olfactory behavior in offspring independent of the pathogen transfer in those generations. However, the mechanism involved in the transfer of obtained memory through *S.* Typhi training to P0 for the avoidance till F3 generations is yet to be known.

### Inherited information confers resistance against the pathogen in the offspring generations

**Fig 5:**
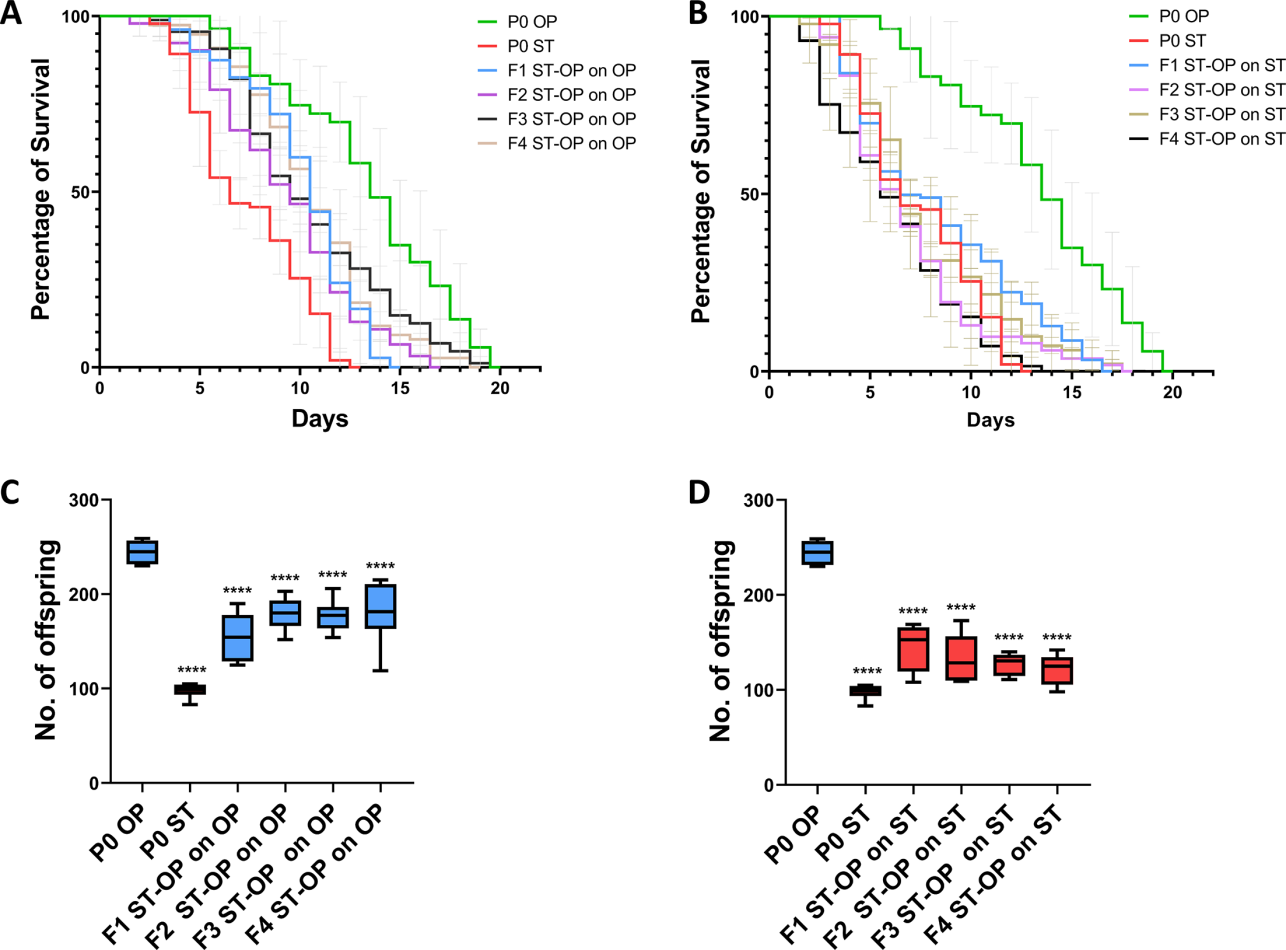
Enhanced resistance against *S.* Typhi in the offspring generations of the P0 *S.* Typhi-trained parents. Transgenerational survival analysis of the offspring generations of the P0 *S.* Typhi-trained parent *C. elegans* on *E. coli* OP50 (**A**) and on *S.* Typhi (**B**), n = ~90 worms. Brood size analysis of the offspring generations of the *E. coli* OP50 fed and *S.* Typhi-trained P0 parent *C. elegans* on *E. coli* OP50 (**C**) and on *S.* Typhi (**D**), n = ~45 worms, (Blue color corresponds to experimentation on *E. coli* OP50 and red color corresponds to experimentation on *S.* Typhi). For statistical analysis for brood size assays, one-way ANOVA (Dunnett’s multiple comparisons test) for multiple comparisons with P0 ST as the control set was carried out (*P* < 0.05).

To investigate whether the inherited information of the parental training could alter the health span of the offspring generations, the survival assays were performed to assess the survivability of the *C. elegans* in parental as well as their offspring generations during infection. Parallelly, we also checked the survivability of the offspring generations (F1–F4) of the P0 *S.* Typhi-trained parent worms upon re-exposure with the same pathogen. Our results show that *C. elegans* survives for ~19 days in *E. coli* OP50 but showed a reduced survivability of ~11 days in *S.* Typhi. The offspring generations of *S.* Typhi-trained P0 generation, that is, F1 ST-OP and F2 ST-OP showed a reduction in the lifespan upto ~14 and ~16 days, respectively, in *E. coli* OP50, which was 26% and 15% lower than the survival of P0 *E. coli* OP50-fed worms, whereas the later generations F3 ST-OP and F4 ST-OP showed an increase in survival upto ~19 and ~18 days, respectively, under *E. coli* OP50 condition (Fig. 5A). This suggests that the parental exposure affected the survivability in the F1 and F2 generations. Interestingly, when the offspring generations of P0 trained in *S*. Typhi were assessed for their survivability in *S.* Typhi, an increase in the survival rate was observed. The survival of F1 ST-OP, F2 ST-OP, and F3 ST-OP in *S.* Typhi was ~16, ~17, and ~17, respectively, which was 45%, 54%, and 54% higher than the survival of parental *C. elegans* on *S.* Typhi, respectively (Fig. 5B). However, on F4 ST-OP, the survival duration reduced to ~13 days. The analysis suggests characteristic short-term enhanced survivability against the candidate pathogen in F1–F3 generations of pathogen trained P0 *C. elegans*. In addition, the reproductive fitness was analyzed through brood size assay. *S.* Typhi infection has been reported to reduce the progeny count in *C. elegans* (46). Our findings suggested that the egg-laying capability in the offspring of *S.* Typhi-trained P0 parents was observed to be reduced when the offspring were maintained on *E. coli* OP50 lawn. The brood size of F1 ST-OP, F2 ST-OP, F3 ST-OP, and F4 ST-OP on *E. coli* OP50 was found to be 36%, 26%, 27%, and 26% lower than the P0 *C. elegans* on *E. coli* OP50, respectively. When checked on *S.* Typhi lawn the brood size of F1 ST-OP, F2 ST-OP, F3 ST-OP, and F4 ST-OP was observed to be 48%, 36%, 30%, and 24% higher than the egg count of P0 *S.* Typhi parent (Fig. 5C and D). The survival and the reproductive assessment showed that although the offspring of *S.* Typhi-trained P0 *C. elegans* showed a decrease in the healthspan parameters, they exhibited enhanced characteristic resistance when re-exposed to the same pathogen.

### Parental infection induces immune priming of offspring mediated through DAF-2/DAF-16 dependent insulin/IGF-1 signaling pathway

**Fig 6:**
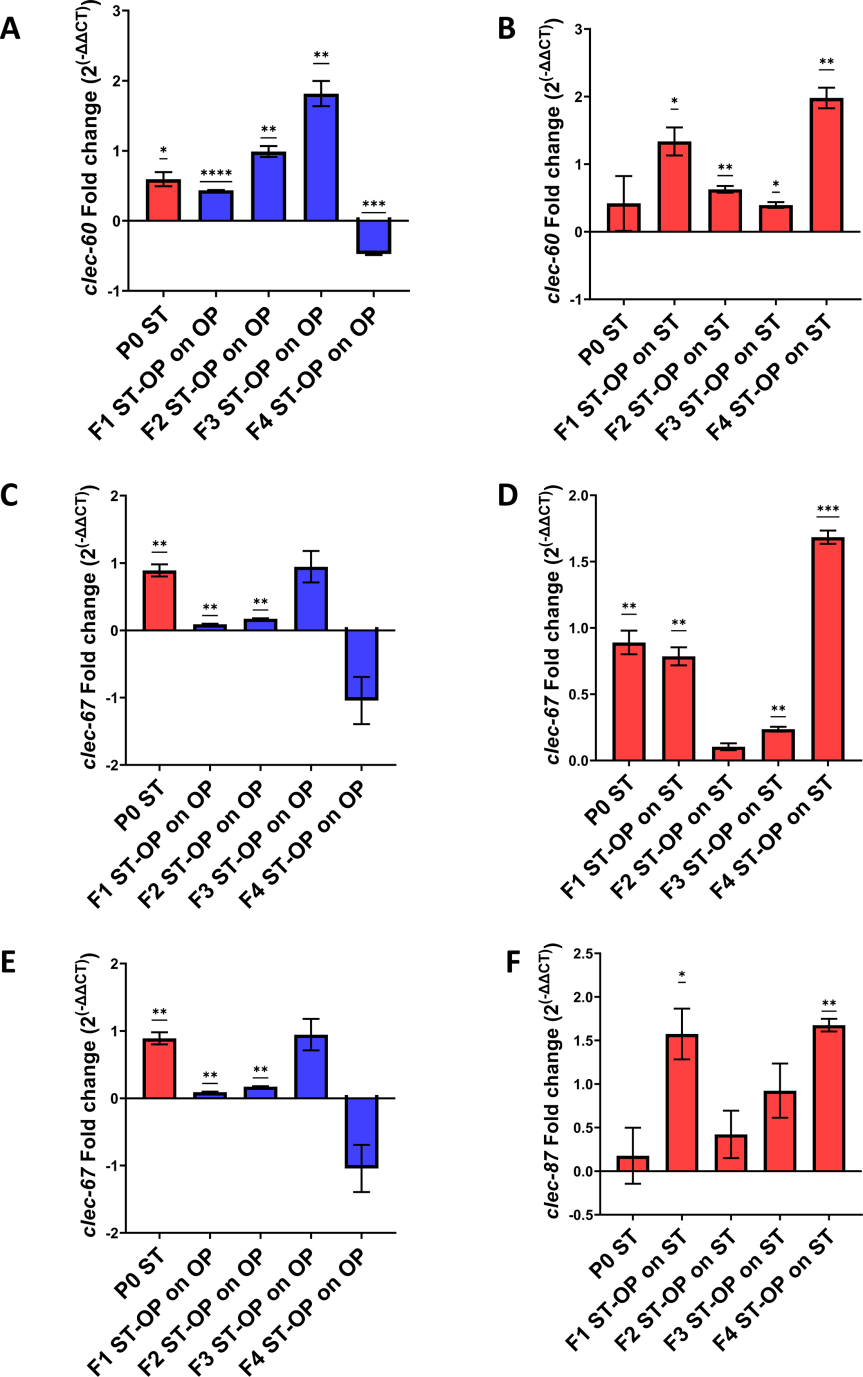
Enhanced expression of C-type lectin genes involved in pathogen recognition and defense response against infection. Relative gene expression of C-type lectin genes in *C. elegans* namely *clec-60*, *clec-67,* and *clec-87* in *S.* Typhi-infected P0 parent and in offspring generations when grown on *E. coli* OP50 (A, C, and E) and when re-exposed to *S.* Typhi (B, D, and F) transgenerationally. For statistical analysis for qPCR, one-way ANOVA (one sample t test) was carried out (*P* < 0.05).

Furthermore, gene expression patterns of candidate innate immunity-o F3 generations of offspring nematodes grown on E. coli related genes were assessed transgenerationally. Increase in the expression of C-type Lectin genes (*clec-60*, *clec-67,* and *clec-87*), which are responsible for pathogen recognition and generating defense response (27), was observed up to F3 generations of offspring nematodes grown on *E. coli* OP50 after P0 parental infection (Fig. 6A, C, and E). In offspring nematodes, which were re-expose to *S.* Typhi, their expression was upregulated up to F4 generations (Fig. 6B, D, and F). This suggests that the parental infection with *S.* Typhi could prime the subsequent generations with increased immunity, which could possibly help in conferring resistance against *S.* Typhi upon re-exposure.

**Fig 7:**
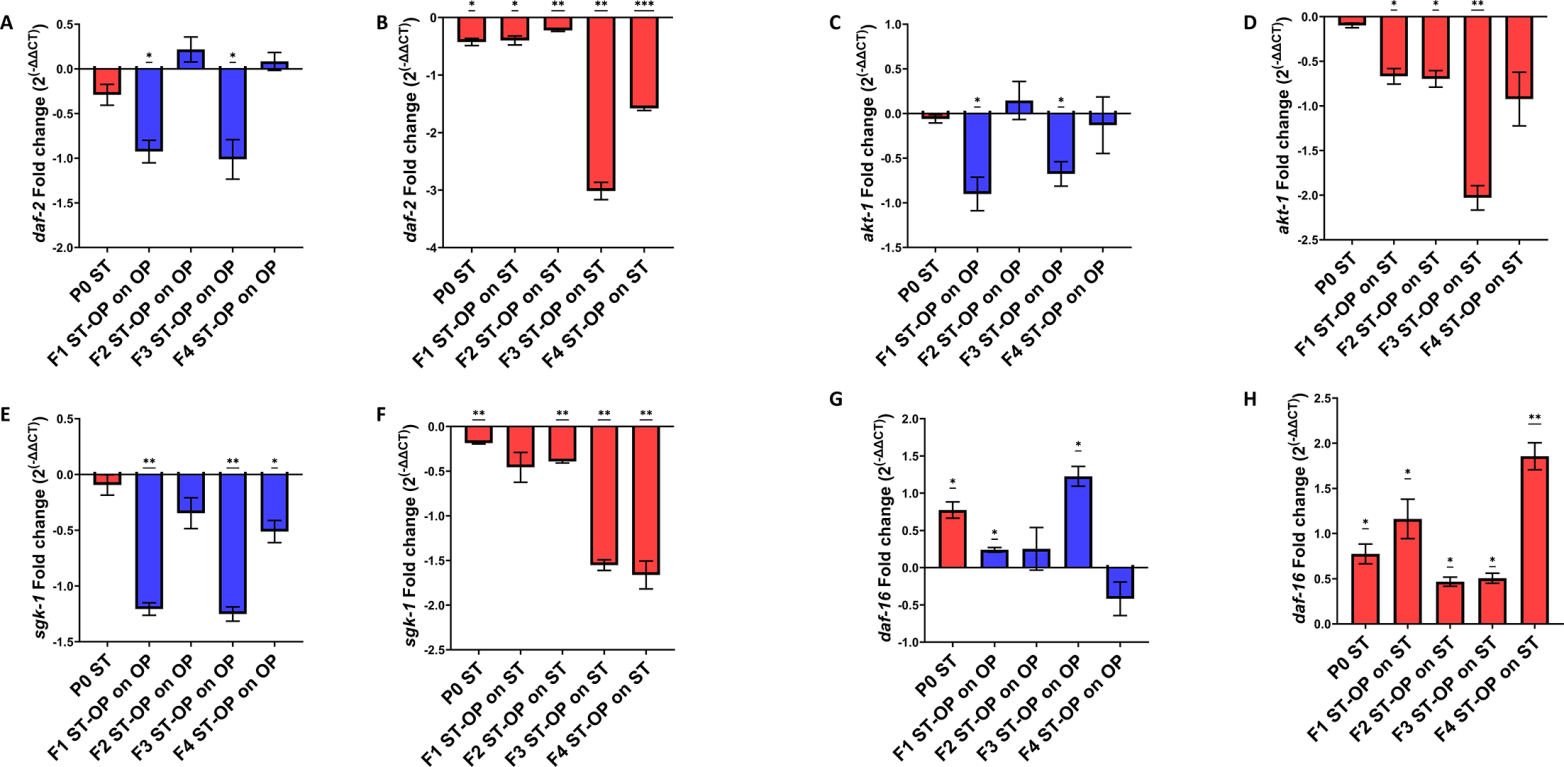
Increased insulin signaling levels could have a role in enhanced resistance against *S.* Typhi. Relative gene expression of genes involved in insulin/IGF-1 signaling pathway, *daf-*2, *akt-*1, *sgk-1,* and *daf-16* in *S.* Typhi-infected P0 parent and in offspring generations when grown on *E. coli* OP50 (A, C, E, and F) and when re-exposed to *S.* typhi (B, D, G, and H) transgenerationally. For statistical analysis for qPCR, one-way ANOVA (one sample t test) was carried out (*P* < 0.05).

Earlier studies suggested that the insulin/IGF-1 signaling pathway has been involved in conferring resistance against pathogenic infections (16, 47). To check if the increased resistance of the offspring generations against *S.* Typhi was mediated through ILS, the level of expression of genes (*daf-2*, *akt-1*, *sgk-1,* and *daf-16)* involved in the pathway was analyzed. The observations showed decreased levels in the expression of *daf-2*, *akt-1,* and *sgk-1.* However, an increase in the expression levels of *daf-16* in the offspring generations when re-exposed to *S.* Typhi was observed (Fig. 7A through H). The findings suggest that the immune priming of the offspring and the enhanced resistance towards *S.* Typhi might be dependent on the DAF-2/DAF-16-mediated insulin signaling pathway.

### Dopamine signaling is essential for the transmission of the learnt information transgenerationally

**Fig 8:**
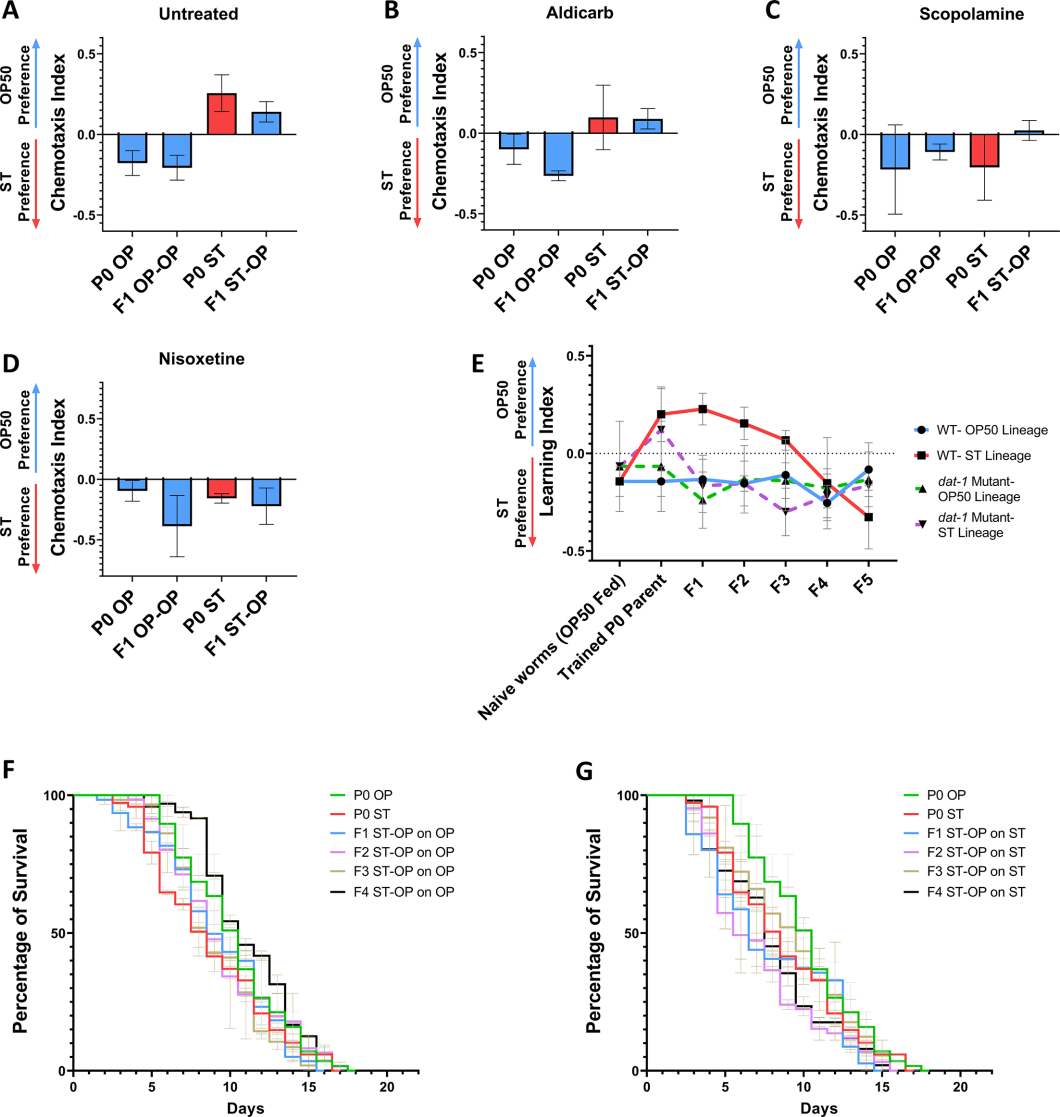
Dopamine signaling is essential for the transmission of learnt information transgenerationally. Olfactory preference of parental *C. elegans*-trained on *S.* Typhi and their F1 offspring upon prior treatment with neuronal modulatory chemicals such as (**B**) Aldicarb, an acetylcholinesterase inhibitor, which causes an increased accumulation of acetylcholine in the synaptic cleft of the neuromuscular junction, (**C**) Scopolamine, which causes increased serotinin levels and (D) Nisoxetine, which inhibits dopaminergic signaling by exhibiting antagonistic activity against DAT-1 (Dopamine transporter), when compared with the untreated control (**A**) *n* = ~450 worms, (Blue color corresponds to *C. elegans* grown on *E. coli* OP50 before experimentation and red color corresponds to *C. elegans* grown on *S.* Typhi before experimentation). (**E**) Transgenerational olfactory preference analysis of *dat-1* mutant *C. elegans*, *n* = ~450 worms. Survival analysis of *dat-1* mutant *C. elegans* (parental and offspring generations) in *E. coli* OP50 (**F**) and upon re-exposure with *S.* Typhi (**G**), n = ~90 worms.

Pathogenic avoidance is a vital defense strategy to evade harmful bacteria, which is mediated by various neuronal signalings. Through these signaling pathways, the animals can sense and learn about the environment (48). As alteration in the olfactory behavior was observed, we wanted to check if the observed transmission of the learnt information was mediated through any neuronal signaling. Prior to the *S.* Typhi training, the parent worms were exposed to selective neuromodulatory molecules. Mammalian norepinephrine transporter inhibitor, Nisoxetine, was reported to disrupt dopamine signaling by exhibiting antagonistic properties in *C. elegans* dopamine transporter, DAT-1, thereby reducing dopamine uptake (31). Scopolamine is a muscarinic antagonist, which could mimic the effects of selective serotonin reuptake inhibitor (SSRI), thereby causing increased levels of serotonin (32). Aldicarb is an acetylcholinesterase inhibitor, which causes an increased accumulation of acetylcholine in the synaptic cleft of the neuromuscular junction, thereby altering the cholinergic pathway (33, 34). Upon exposure to these neuromodulatory molecules, the avoidance behavior of the *S.* Typhi-trained P0 and their immediate F1 ST-OP offspring was assessed using chemotaxis assays. Aldicarb-treated P0 and their offspring showed no altered behavior when compared with the control untreated *C. elegans* (Fig. 8A and B). Scopolamine-treated P0 parent worms did not exhibit pathogenic avoidance, but a slight avoidance was observed in their F1 ST-OP offspring worms, which was negligible (Fig. 8C). The worms that were pre-treated with Nisoxetine and their offspring did not exhibit avoidance toward *S.* Typhi upon pathogen training. This could possibly mean that healthy and functional dopamine signaling is essential for the worms to generate a characteristic olfactory response toward a pathogen. In the absence or alteration of the signaling, the adaptive trait was neither observed in the parent nor was transmitted to the offspring (Fig. 8D). These results suggested that active dopamine signaling is essential for *C. elegans* to learn and exhibit a heritable avoidance toward the pathogen *S.* Typhi.

To validate our findings, *dat-1* mutant *C. elegans,* which has loss of function of dopamine transport, was used for experimentation. Learning index was assessed transgenerationally upon training the P0 parent worms in *S.* Typhi. Interestingly, it was observed that the P0 parent worms trained on the pathogen showed avoidance, which was not observed in their subsequent generations as seen in wild-type *C. elegans.* (Fig. 8E). This suggests that although avoidance of the parent worms due to learning takes place within the generation, dopamine signaling is required for the transmission of the learnt information across generations. Furthermore, we also checked if the *dat-*1 mutants show pathogenic resistance as exhibited by the offspring generations in the wild-type worms. Contrary to our observation in the wild-type *C. elegans,* the *dat-1* mutants survived better in *S.* Typhi and showed a survival of ~16 days, whereas in *E. coli* OP50, they showed a survival of ~17 days. The survivability of the offspring generations in *E. coli* OP50 was as follows: F1 ST-OP on OP: ~15 days, F2 ST-OP on OP: ~17 days, F3 ST-OP on OP: ~14 days, and F4 ST-OP on OP: ~16 days (Fig. 8F). Similarly, their survival in *S.* Typhi were as follows: F1 ST-OP on ST: ~16 days, F2 ST-OP on ST: ~14 days, F3 ST-OP on ST: ~15 days, and F4 ST-OP on ST: ~15 days (Fig. 8G). These results show that unlike wild-type worms, the offspring generations of the P0 *S.* Typhi-trained *dat-1* mutants did not show resistance against the pathogen. Cumulatively, the findings suggest the importance of dopamine signaling in the transmission of information in the parental environment and in generating an inherited adaptive response and enhanced resistance against the pathogen.

### Parental *S.* Typhi training modulates dopaminergic signalIng players in the offspring generations

**Fig 9:**
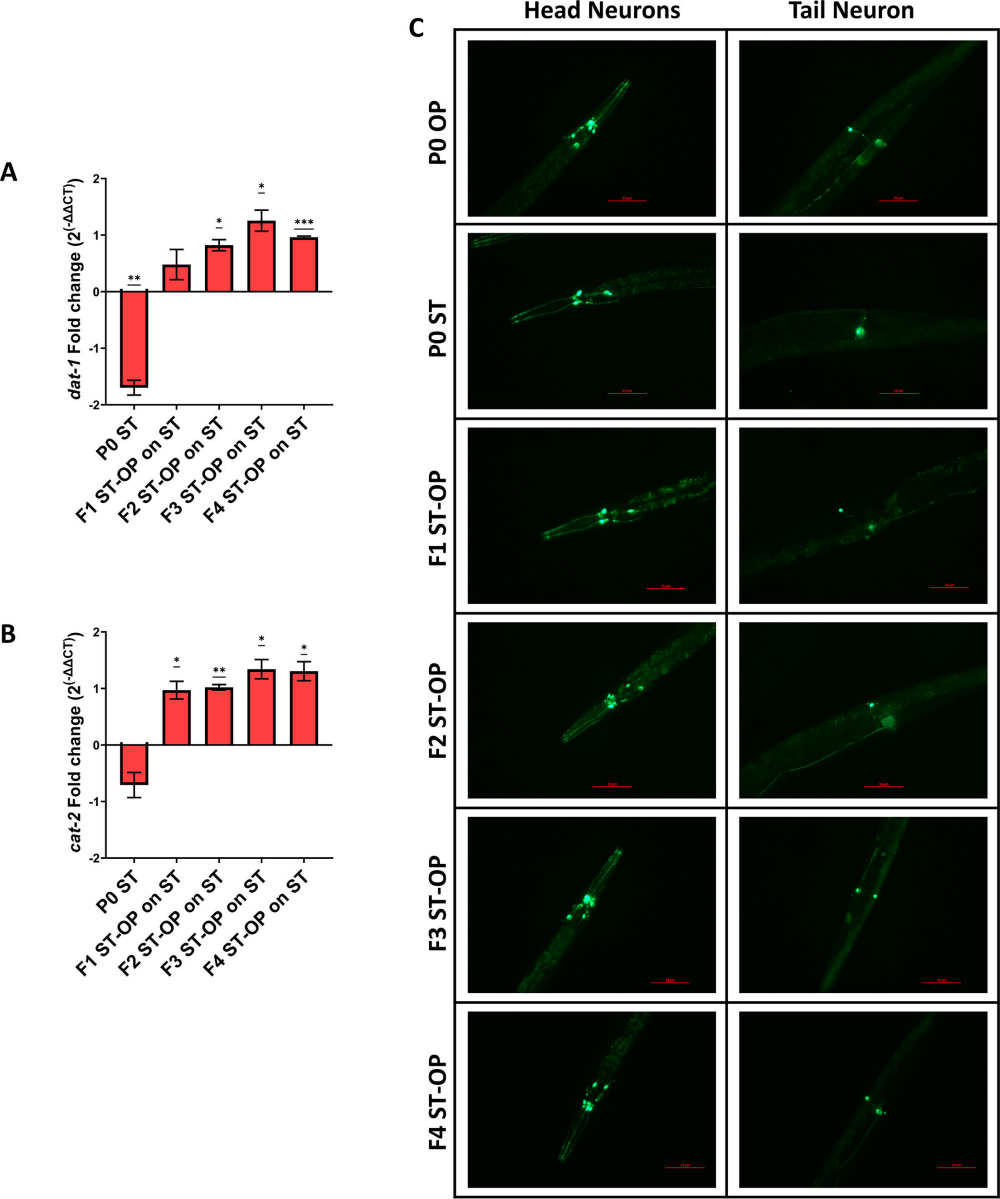
Enhanced dopamine signaling upon re-exposure with *S.* Typhi transgenerationally. Relative gene expression of *dat-1* (dopamine transportation and re-uptake) and *cat-2* (dopamine biogenesis) in *S.* Typhi-trained parent *C. elegans* and their subsequent offspring generations when they were re-exposed with *S.* Typhi (A and B) (Scale bar: 25 µm). For statistical analysis for qPCR, one-way ANOVA (one sample t test) was carried out (*P* < 0.05). (C) Modulations in the *in vivo* localization of DAT-1 expressed in dopaminergic neurons (CEP and ADE in the head region and PDE in the tail region) across generations of P0 *S.* Typhi-trained *C. elegans* upon re-exposure with the pathogen.

To further investigate the role of dopamine signaling in the transgenerational avoidance behavior, the role of dopamine signaling regulating players was assessed using gene expression studies and *in vivo* imaging. The mRNA levels of *dat-1* (dopamine transport) and *cat-2* (Dopamine biogenesis) were investigated. In the parental worms trained with *S.* Typhi, the expression levels of both *cat-2* and *dat-1* were found to be downregulated. Strikingly, when these offspring generations were re-exposed to *S.* Typhi, the levels of both *dat-1* and *cat-2* were found to be significantly upregulated in the offspring generations (Fig. 9A and B). Next, to check the *in vivo* expression of dopamine signaling, we used a p*dat-1*::GFP (BZ555) *C. elegans. C. elegans* consists of eight dopaminergic neurons distributed into three types (CEP, ADE, and PDE). Upon training of P0 worms on *S.* Typhi, we observed a modulated expression of *dat-1* in the dendrites of CEP and ADE head neurons and reduced expression in the PDE tail neurons, which supported the modulation of *dat-1* mRNA levels. Alterations were also observed in the form of higher expression in the offspring generations, suggesting the inheritance of neuronal signaling modifications across generations (Fig. 9C). Enhanced expression upon re-exposure in the offspring generations might help the host generate learnt adaptive response against the same pathogen. Together, these results suggest an increased pattern in the expression of dopaminergic signaling players in the offspring could help the offspring learn from the environment and generate an adaptive response.

### *C. elegans* shows an adaptive preference for *S.* Typhi upon multigenerational pathogenic training

**Fig 10:**
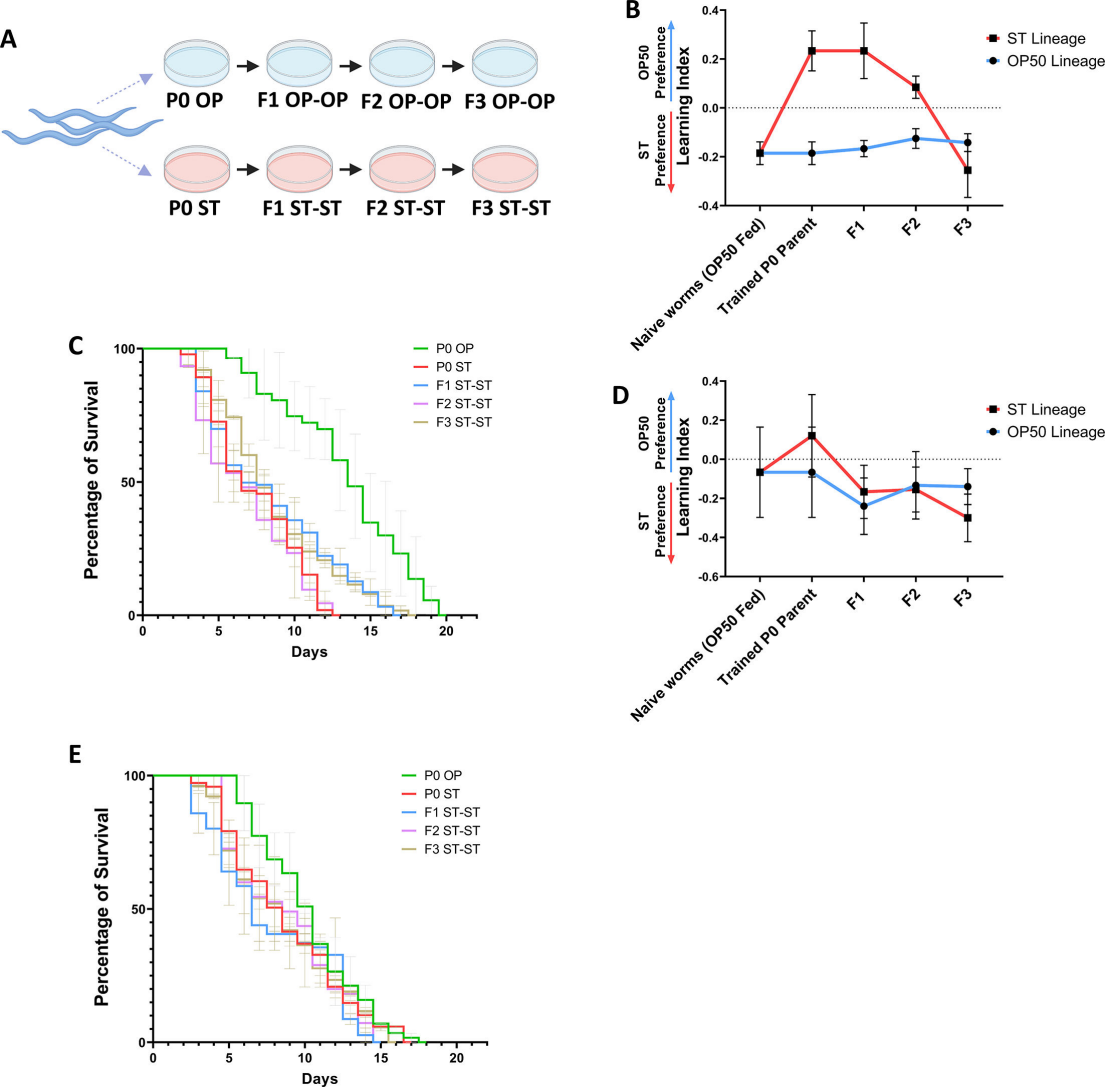
*C. elegans* exhibit adaptive preference upon multigenerational *S.* Typhi training against the pathogen. (**A**) Schematic representation of multigenerational experimental setup where the P0 parental generation and their subsequent offspring generations are trained in *S.* Typhi (Blue color plate denotes *C. elegans* grown on *E. coli* OP50 and red color denotes *C. elegans* infected with *S.* Typhi). (**B**) Olfactory learning index upon multigenerational *S.* Typhi training in wild-type *C. elegans*, *n*= ~450 worms. (**C**) Multigenerational survival analysis of wild-type *C. elegans* on *S.* Typhi, n = ~90 worms. (**D**) Olfactory learning index upon multigenerational *S.* Typhi training in *dat-1* mutant *C. elegans*, (Blue line corresponds to the lineage of *E. coli* OP50 fed worms and red color corresponds to the lineage of *S.* Typhi-trained worms), *n*= ~450 worms. (**E**) Multigenerational survival analysis of *dat-1* mutant *C. elegans* on *S.* Typhi, n = ~90 worms.

**Fig 11:**
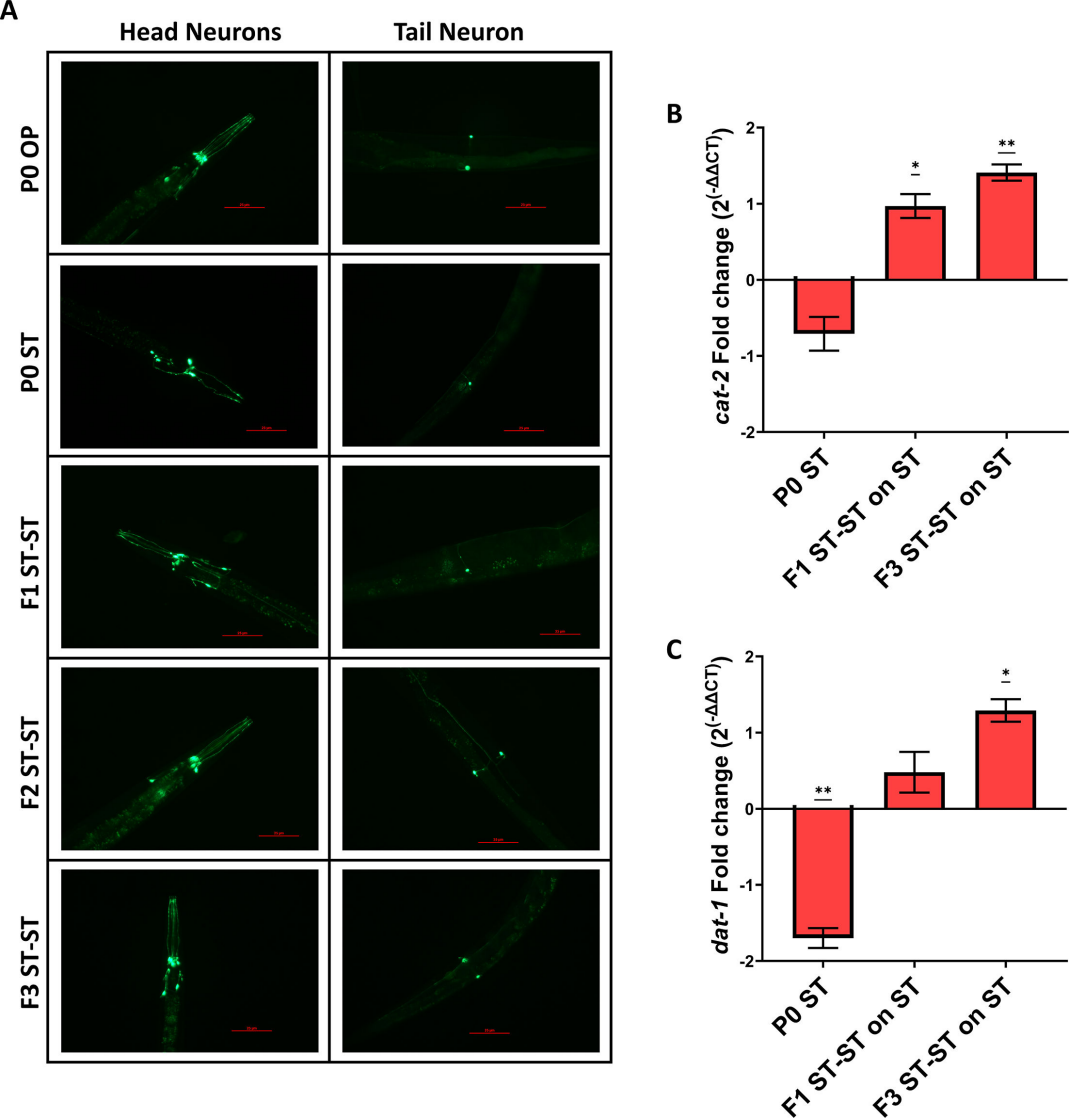
Molecular modulations of dopamine signaling upon multigenerational *S.* Typhi training. (**A**) Differential modulations in the *in vivo* localization of DAT-1 expressed in dopaminergic neurons (CEP and ADE in the head region and PDE in the tail region) when *C. elegans* (BZ555) were trained on *S.* Typhi multigenerationally (Scale bar: 25 µm). Relative gene expression of *cat-2* and *dat-1,* suggests that multigenerational exposure of three generations to *S.* Typhi could enhance the expression of dopamine signaling players (B and C). For statistical analysis for qPCR, one-way ANOVA (one sample t test) was carried out (*P* < 0.05).

**Fig 12:**
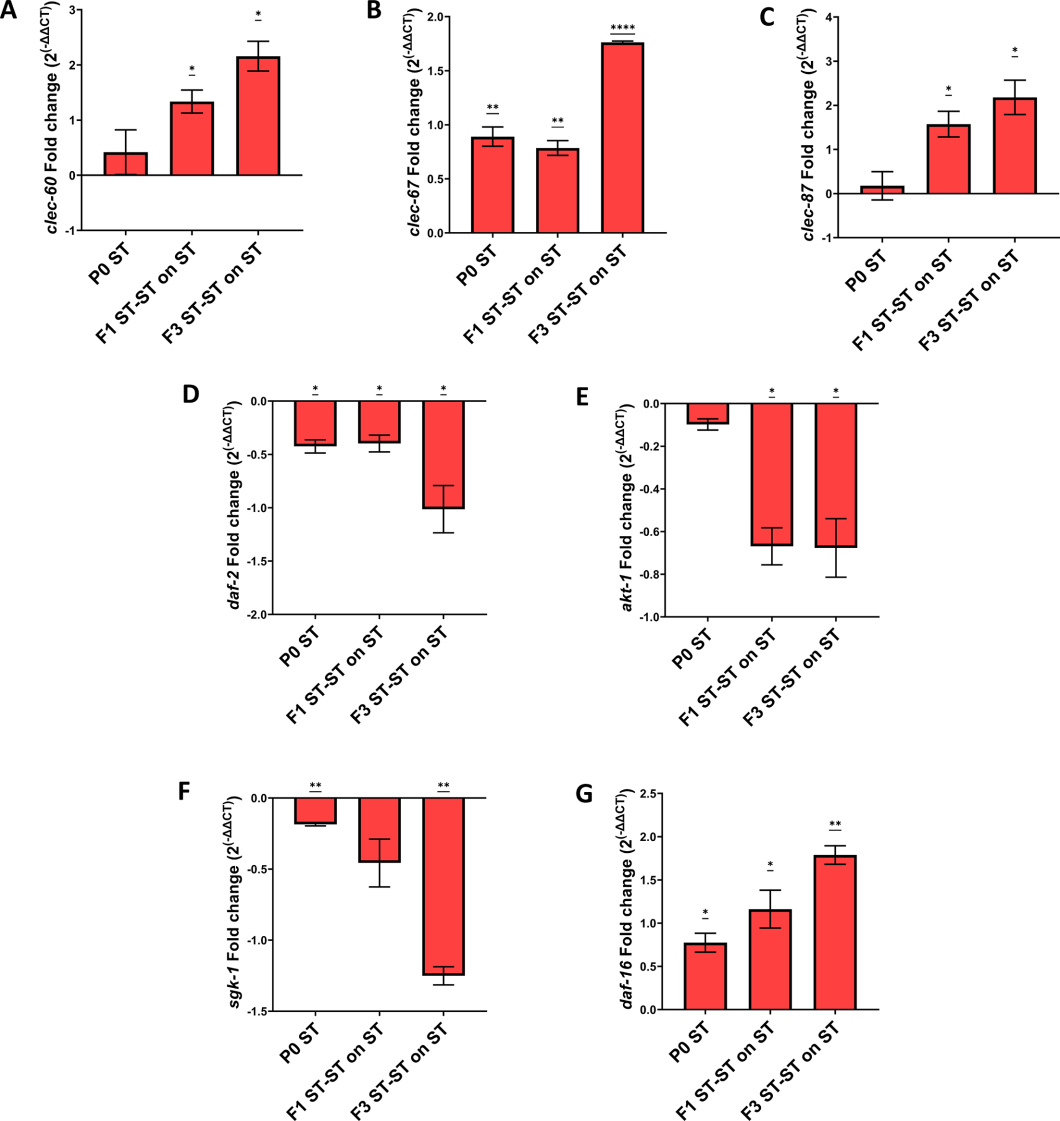
Higher levels of immune-related genes and pathway upon multigenerational *S.* Typhi training. Relative gene expression of C-type lectin genes *clec-60*, *clec-67,* and *clec-87* (A, B, and C) and insulin signaling pathway, *daf-*2, *akt-*1, *sgk-1,* and *daf-16* (D, E, F, and G) upon multigenerational training on *S.* Typhi. For statistical analysis for qPCR, one-way ANOVA (one sample t test) was carried out (*P* < 0.05).

As we observed how a single generation parental training of *C. elegans* to the pathogen *S.* Typhi could induce learnt avoidance toward the pathogen, which could be inherited across generations, our next question was to check the response of the worms if they are trained on the pathogen for a particular duration at every subsequent generation. For this, the parental P0 and offspring generations F1 ST-ST and F2 ST-ST were continuously trained on the pathogen for 24 h. Surprisingly, when the next generation F3 ST-ST was checked for their preference upon pathogen training, they started preferring the pathogen (Fig. 10A and B). The findings suggest that the worms that showed a learnt avoidance toward the pathogen could develop an adaptive response and start preferring the pathogen after F3 generations of pathogenic training. Next, we wanted to check the survival capability of the F3 ST-ST generation worms after multigenerational pathogen training. We observed that the parental P0 worms survived for ~11 days in *S.* Typhi, whereas the F3 ST-ST generation worms survived for ~17 days in the same pathogen (Fig. 10C). The findings suggest that upon four consecutive generations of *S.* Typhi infection, the worms could develop an adaptive response against the pathogen which could help them survive better and also start accepting the pathogenic bacteria as a food source. To check the role of dopamine signaling in the adaptive response, olfactory modulation was also assessed for the *dat-1* mutant worms. *dat-1* mutant worms did not exhibit avoidance of the pathogen beyond parental generation and started preferring the pathogen as early as F1 generation (Fig. 10D). Hence, a survival assay was carried out to check if this preference was similar to that of the adaptation observed in wild-type worms. Multigenerational training of *dat-1* mutant animals did not exhibit enhanced survivability at any of the observed offspring generations (Fig. 10E). *In vivo* p*dat-1*::GFP expression also suggested modulation in the dopamine signaling patterns and was subsequently validated at the mRNA levels of *dat-1* and *cat-2,* which also suggested a significantly increased dopamine biogenesis and transportation upon multigenerational exposure (Fig. 11A through C). Further analysis reveals a significant enhancement in the expression of C-type Lectin genes and enhanced levels of insulin/IGF-1 signaling pathway-related genes which suggested that the exhibited adaptive behavior could probably be mediated through insulin signaling (Fig. 12A through G).

## DISCUSSION

The ability of an organism to learn from an environment and associate the learning to generate an adaptive response, which could alter phenotypical state of the organism has been conserved (49). The experiences help the animals to learn and develop aversive olfactory responses toward the pathogen that could probably be adaptive in nature (44). Previous works in our lab using *C. elegans* to study *S*. Typhi infection showed that the worms could not generate avoidance of *S.* Typhi despite its pathogenic nature (27). The role of immune regulatory players was also assessed to study the hosts’ innate defense in response to the pathogenesis (35). However, the long-term impact of pathogenesis in host modulation toward the infection has not been addressed. Here, we show that *S.* Typhi infection at parental generation could induce behavioral, physiological, and molecular alterations, which are inherited across multiple generations. The offspring have survived better when exposed to the same pathogen, which is probably mediated by the selective neuronal and immune signaling pathways.

Initially, we found that *C. elegans* upon feeding the candidate pathogen *S.* Typhi in a solid bacterial lawn in the form of training showed a characteristic avoidance of *S.* Typhi, which was in accordance with the previous report which suggested *C. elegans* could develop avoidance to an attractive pathogen upon infection (44). Also, the trait was observed after 24 h of training and not at lower durations like 2 and 12 h, suggesting that an extended duration of colonization is required to exhibit a modulated behavioral learning pattern. Although it was previously reported that parental stress response could generate heritable modulation, we checked the olfactory of the F1 offspring worms of the *S.* Typhi-trained parents that were grown in *E. coli* OP50 (24, 50). We found that the avoidance behavior toward *S.* Typhi was also seen in F1 worms, suggesting that the modulations could be heritable across generations from the diseased parent to its offspring. The avoidance behavior was also specific toward *S.* Typhi as neither the P0 *S.* Typhi-trained parents nor their F1 offspring exhibited modulated olfaction when tested against another gram-negative pathogen *V. alginolyticus* or a probiotic bacterium *L. plantarum.*

Bacterial infection is characterized by intestinal damage requiring due to pathogen colonization, which has also been reported to be involved in pathogen avoidance behavior (51). We observed an accumulation of eggs in the P0 *S.* Typhi-trained parent and its F1 offspring and disruption in the intestinal lumen along in the P0 *S.* Typhi-trained parent, which made us question if there was a transmission of the pathogen to the next generations? Earlier report from our lab found that the presence of pathogen in F1 offspring from the infected worms (45). The reduced bacterial load in the offspring could be due to the clearance of the pathogen by the activated innate defense response in against the pathogen (52). The pathogenic avoidance behavior exhibited upon infection could last for several generations and this decision-making ability of the animals is mediated by epigenetic mechanisms (53). In our study, the avoidance toward *S.* Typhi upon parental training persisted upto three subsequent offspring generations. However, the pathogen colonization in the worm was observed with only one offspring generation. Hence, although intake of the bacterium and colonization is essential for the induction of infection and generation of avoidance behavior toward the pathogen, there could be other regulating mechanisms that could possibly be playing the role of maintaining the inherited state transgenerationally.

Furthermore, the present study monitored if the inherited state has an effect in the fitness of the organism in terms of their survivability. We observed that the infection reduced survival rates in the P0 parental worms and their offspring generations that were grown in non-pathogenic *E. coli* OP50. Interestingly, these offspring worms exhibited enhanced survival when grown in *S.* Typhi. This was in contrast with the previous findings stating that there was no significant variation in the survivability of the offspring upon parental infection with *S. enterica* (54). C-type Lectin proteins have been reported to be involved in pathogen recognition and generating defense against the invading pathogen upon infection. Increased expression of *clec-60*, *clec-67,* and *clec-87* in the infected parent and offspring generations suggests that the parental experience could induce enhanced immunity in the offspring, which could help in their enhanced survival upon re-exposure. This shows that the response could be specific depending on the pathogen on which the parent was trained on. More studies are required to understand this phenomenon clearly.

Insulin/IGF-1 signaling (IIS) has been reported to be involved in conferring resistance against stresses and enhancing lifespan associated parameters in *C. elegans* (55–57). This is regulated by insulin-like peptide ligands that bind to DAF-2, which is an ortholog of insulin/IGF-1 transmembrane receptor (IGFR). Reduced/depleted expression of *daf-2* has been previously reported to be involved in conferring resistance against pathogenic infections by activating the DAF-16/Forkhead box O transcription factor (58). In the present study, downregulation in the expression of *daf-2* and its downstream signaling players *akt-1* and *sgk-1* mRNA levels and increased expression of *daf-16* was observed transgenerationally suggesting that the increased resistance against *S.* Typhi in the offspring generations could probably be mediated by IIS.

*C. elegans* protect themselves from the deleterious effects of infection with the help of multiple signaling pathways mediated by numerous sensory neurons, as they are involved in pathogenic avoidance (59). Hence, we wanted to check if the observed heritable avoidance behavior toward *S.* Typhi is mediated by neuronal signaling. We modulated selective neuronal signaling *C. elegans* with the help of modulatory chemicals such as Nisoxetine, which inhibits dopamine transportation, Scopolamine, which causes increase in serotonin levels, and Aldicarb, which is an inhibitor of acetylcholinesterase in cholinergic signaling. We found that pre-treatment with Nisoxetine inhibited the avoidance behavior upon *S.* Typhi training, which was subsequently validated by the experiments using dopamine transportation (*dat-1*) mutant *C. elegans.* Dopamine, in addition to being major player involved in reward pathways, is also involved in modulating immune responses as numerous immune cells express dopamine receptors, conferring the aspect of dopaminergic immunomodulation (60, 61). Also, the offspring generations of the *S.* Typhi-trained P0 parent *C. elegans* did not exhibit enhanced survival, suggesting that the dopaminergic signaling might be essential for the transgenerational adaptive behavior. Learning and behavioral changes in *C. elegans* might be controlled by dopaminergic neurons and (22) stated that mechanosensory alterations in CEP and ADE neurons might contribute to stress-induced aversive learning. Modulations in the *in vivo* expression of *dat-1* in CEP, ADE neurons in the head region, and PDE neurons in the tail region in the P0 *S.* Typhi-trained parent and in their subsequent offspring generations was observed. Also, the mRNA levels of *dat-1* and *cat-2* involved in dopamine reuptake and synthesis were also found to be altered. Reference (22) stated that mutated expression of *cat-2* and *dat-1* could impair the learning in *C. elegans*. In this context, the present study suggested that bacterial infections can alter dopaminergic signaling that are heritable across generations and have a role in inducing learnt pathogenic avoidance and could prime the animal’s fitness upon parental training to *S.* Typhi.

As a single parental exposure in *S.* Typhi was found to prime the offspring with enhanced resistance against the pathogen, we wanted to know the effects of long-term (multigenerational) pathogenic exposure. Multiple generational exposures of *C. elegans* with *P. vranovensis* has been reported to generate adaptation with enhanced survivability (62). Hence, we wanted to monitor the response when the *C. elegans* were trained on the *S.* Typhi lawn for duration of 24 h at every subsequent generation. Interestingly, the worms which initially showed avoidance to *S.* Typhi, started exhibiting preference to the pathogen after three generations of long-term (multigenerational) training (F3 ST-ST). We presumed that the preference to *S.* Typhi could be an adaptive way of accepting it as food source. Hence, when we checked their survivability against the pathogen, we observed that the F3 ST-ST worms survived better on *S.* Typhi than the initial parental exposed worms. This adaptive phenotype was not observed when *dat-1* mutant animals were tested in a similar manner. Even when exposed continuously, expression of dopamine signaling players was increased in the adapted generation, suggesting the role of dopamine signaling in the adaptive response. Also, enhanced expression of C-type lectin genes and insulin signaling was observed in the adapted generation worms, which suggested that the adaptive response response could be mediated through insulin signaling.

The nervous system plays a multifaceted role during bacterial infections as it can either activate and/or suppress the defense mechanism against the infections (48). In the present study, we observed that the adaptive response toward the pathogen was exhibited upon *S.* Typhi training, and these alterations were inherited across generations. Furthermore, this transiently modulates the host’s olfactory behavior that subsequently rendering resistance to the host against the pathogen and enhancing their survivability. The reduction in dopamine signaling in the *S.* Typhi challenged P0 worms and higher levels in the resistant offspring suggest that it could be playing the role in rendering protection. This provides a new aspect of the functioning of the dopaminergic signaling in the transmission of the memory across generation apart from the already known role like learning and reward system. Overall, the present data propose a mechanism by which how a bacterium can influence the parental host and its subsequent generations, impacting behaviors related to pathogen avoidance and potentially enhancing evolutionary fitness. However, it remains to be understood as to how these modulations are passed on to the offspring generations. A recent work from our lab stated that bacterial infections might alter the metabolomic profiles pertaining to neuro-immune signaling (38). Also, the alterations in the metabolite expression are specific to the pathogenic infection (63). As metabolites are key molecules in regulating the signaling between the intestine and the nervous system, they could possibly have a role in priming the host’s response and also could be involved in transmission of the learnt information across generations, which needs to be assessed in future studies. In particular, detailed studies are required to address how a specific metabolite can interact with signaling pathways to generate inheritance and adaptive behavior.

## Data Availability

Data from the current study are available from the corresponding author on reasonable request.
